# Involvement of cyclin B1 in progesterone-mediated cell growth inhibition, G2/M cell cycle arrest, and apoptosis in human endometrial cell

**DOI:** 10.1186/1477-7827-7-144

**Published:** 2009-12-07

**Authors:** Li Tang, Yu Zhang, Hong Pan, Qiong Luo, Xiao-Ming Zhu, Min-Yue Dong, Peter CK Leung, Jian-Zhong Sheng, He-Feng Huang

**Affiliations:** 1 Department of Reproductive Endocrinology, Women's Hospital, School of Medicine, Zhejiang University, Hangzhou, China; 2 Department of Pathology and Pathophysiology, School of Medicine, Zhejiang University, Hangzhou, China; 3 Department of Obstetrics and Gynaecology, Child and Family Research Institute, University of British Columbia, Vancouver, British Columbia V6H 3V5, Canada; 4 Department of Reproductive Endocrinology, The First People's Hospital of Yunnan Province, Yunnan, China

## Abstract

**Background:**

Progesterone plays an important role in the proliferation and differentiation of human endometrial cells (hECs). Large-dose treatment with progesterone has been used for treatment of endometrial proliferative disorders. However, the mechanisms behind remain unknown.

**Methods:**

To investigate the role of cyclin B1 in proliferation and differentiation of hECs in menstrual cycle, the expression of cyclin B1 throughout the menstrual cycle was evaluated in hECs. To determine the effects of progesterone on the proliferation, cell cycle progression and apoptosis of hECs and to test if cyclin B1 is involved in these effects, progesterone and/or Alsterpaullone (Alp, a specific inhibitor of Cyclin B1/Cdc2) were added to primary hECs. Cellular proliferation was evaluated with MTT test, cell cycle with propidium iodide (PI) staining and flow cytometry, apoptosis with FITC-Annexin V and the expression of cyclin B1 with Western blotting.

**Results:**

The expression level of cyclin B1 in secretory endometria was significantly lower than in proliferative endometria (p < 0.01). Progesterone significantly inhibited the growth of hECs in a concentration-dependent manner (P < 0.01). The treatment with progesterone significantly decreased the expression of cyclin B1, increased the proportions of cell in G2/M phase, and apoptotic cells (P < 0.05 for all). The presence of Alp significantly enhanced the effects of progesterone on cyclin B1 down-regulation, G2/M cell cycle arrest and induction of apoptosis (P < 0.01 for all).

**Conclusion:**

Our findings suggest that cyclin B1 is a critical factor in proliferation and differentiation of hECs. Progesterone may inhibit cell proliferation, mediate G2/M cell cycle arrest and induce apoptosis in hECs via down-regulating Cyclin B1.

## Background

Progesterone plays a pivotal role in female reproduction. It modifies the effects of estrogen on the endometrium[1]. Estrogen stimulates proliferation of both glandular epithelial cells and stromal cells, whereas progesterone prevents this effect and induces secretory changes in glandular epithelial cells and decidual changes in stromal cells[2]. The balance between these two hormones plays important roles in regulation of the menstrual cycle, ovulation, implantation and pregnancy.

The potent anti-proliferative effect of progesterone has been utilized for treatment of endometrial proliferative disorders[3]. Clinically, progesterone has been used for contraception and the treatment of endometrial hyperplasia and adenocarcinoma as well as endometriosis [4-6]. It is known that long term and large dose treatment with progesterone analogs may lead to the limitation of endometrial growth, atrophy, apoptosis and even cell death[7]. Therapeutic use of progesterone is often associated with irregular and unwanted bleeding[1]. Recent clinical studies have also raised concern about an increased risk of cardiovascular disease or breast cancer[8]. It highlights the importance of insights from molecular biology of progesterone action on endometrium which may provide us with more precise markers for progesterone actions and thus help avoid side-effects and lead to new therapeutic proposal.

Previous studies have shown that progesterone regulates endometrial cell proliferation and differentiation through a nuclear receptor-mediated mechanism, such as down-regulation of estrogen receptor[9,10]. The progesterone-induced growth suppression of endometrial cells has also been explained in various ways such as the elevated activity of steroid metabolizing enzymes[11], growth factors and cytokines[12]. However, the underlying molecular mechanisms by which progesterone negatively regulates the growth of endometrial cells are still not fully understood.

Cell proliferation is restrained through the control of the cell cycle[13]. Cyclin B1 is the key component of the cell cycle machinery[14]. Cyclin B1 binds to Cdc2 at the beginning of G2 phase forming an activated cyclin B1/Cdc2 complex and then phosphorylates its downstream substrates which control the G2 to M transition and promote cell mitotic division[15]. Unscheduled mis-regulation of cyclin B1 during the cell cycle leads to uncontrolled cell growth and aberrant cell function[16]. It is also reported that cyclins are functionally involved in the rhythmic proliferation of normal human endometrial tissue[17]. Moreover, upregulated expression of cyclin B1, cyclin D1 and cyclin E was detected in endometrial carcinomas, which indicated that cyclins might be the major cell cycle regulators involved in endometrial cell proliferation and differentiation[18]. Up to date, it is still unclear whether cyclins are mediated in the negative regulation of the endometrium by progesterone.

As the detection of significantly down-regulated expression of cyclin B1 in secretory endometria strongly suggests that cyclin B1 plays an important role in proliferation and differentiation of hECs under steroids regulation, we then examined the effects of progesterone on the proliferation, cell cycle progression and apoptosis of hECs and tested if cyclin B1 is involved in these effects. In addition, we determined whether Alsterpaullone (Alp, a specific inhibitor of Cyclin B1/Cdc2) is capable of enhancing the effects of progesterone on cyclin B1 down-regulation, G2/M cell cycle arrest and induction of apoptosis.

## Methods

### Subjects

Endometrial tissues were obtained from 18 women (at proliferative phase) who underwent hysterectomy or hysteroscopy and 12 women (at middle secretory phase) who underwent aspiration biopsy during IVF program for benign uterine diseases in Women's Hospital, School of Medicine, Zhejiang University. Written informed consents were obtained from all subjects and the Institutional Review Board of School of Medicine, Zhejiang University, granted the ethical approval for the current investigation. All the women had regular menstrual cycles and received no hormonal treatments three months prior to the operation. Their ages ranged from 29 to 47 years. The menstrual cycle phase was confirmed by histologic dating.

### Cell culture

6 cases of endometrial tissue at proliferative phase were minced in Hanks' solution and digested with 0.2% collagenase (Gibco-BRL, Gaithersburg, MD, USA) at 37°C for 50 min. The dispersed cells were filtered through a 70-mm nylon mesh to remove the undigested tissue pieces. Cells, containing endometrial epithelial cells and endometrial stromal cells were collected and re-suspended in Dulbecco's modified Eagle's medium (DMEM, Gibco-BRL) supplemented with 10% fetal bovine serum (Invitrogen), 50 U/ml penicillin, and 50 μg/ml streptomycin in a 60-mm or 10-mm petri dish or 24 well plate in a humidified atmosphere of 5% CO_2 _at 37°C. The medium was changed every day. Progesterone at the concentration of 1 × 10^-9^, 1 × 10^-8^, 1 × 10^-7 ^or 1 × 10^-6 ^M or progesterone (1 × 10^-7 ^M) and/or Alp (5 mM) (Calbiochem, USA) was added and the cells were incubated for another 72 h before specific experiments. Each experiment was repeated at least three times.

### MTT assay

MTT (3- [4, 5-dimethylthiazol-2-yl]-2, 5-diphenyl tetrazolium bromide) assay was used to evaluate the cellular proliferation. Briefly, after hECs were treated with progesterone for 72 h, 20 μl of MTT (5 mg/ml) (Sigma, USA) was added and the cells were incubated for additional 4 h at 37°C. When the incubation finished, 200 μl of Dimethyl sulfoxide (DMSO) was added and the optical densities (OD) were read at 490 nm with a microplate reader. The experiment was conducted in triplicate and repeated three times. Inhibition rate was calculated as following: (1-OD sample/OD control) × 100%.

### Cell cycle analysis

Cells were detached by trypsinization, washed three times with cold PBS and fixed with 80% ethanol at 4°C for 3 h. For propidium iodide (PI) staining, cells were washed three times with PBS to remove trace ethanol. The pellets were re-suspended and stained with propidium iodide (PI) (Sigma, USA)staining solution (0.1 mg PI and 0.5 mg/ml RNase A in PBS) and incubated at 37°C for 30 min. Cells were analyzed with flow cytometry FACS EPICS (Coulter Epics Altra flow cytometer; Beckman Coulter, Fullerton, CA).

### Detection of apoptotic cells

Cells were detached, washed and re-suspended in 200 μl medium and fluorescently labeled by addition of 20 μl of binding buffer and 5 μl of Annexin V-FITC (Pharmingen, SanDiego, CA). After the incubation at room temperature in dark for 15 min, 2 μl of PI (1 mg/mL, Invitrogen, USA) was added and cells were applied to flow cytometry (Coulter Epics Altra flow cytometer). A minimum of 10,000 cells with in the gated region was analyzed.

### Protein extraction and Western blot analysis

Tissues and cells were washed with PBS and lysed in lysis buffer (1× PBS, 1% Nonidet P-40, 0.5% sodium deoxycholate, 0.1% SDS, 100 μg/ml phenylmethylsulfonyl fluoride, 100 μg/ml leupeptin). The suspension was centrifuged at 15,000 g for 15 min at 4°C, the supernatant was collected and protein concentrations were determined using the Bradford method. 30 μg of protein per lane was loaded and separated on a 10% Sodium dodecylsulfate (SDS)-polyacrylamide gel and transferred to Nitrocellulose Transfer membrane (PROTRAN, BioScience, Germany). Membranes were incubated with blocking buffer (50 mM Tris-HCl, pH 7.6, 150 mM NaCl, 0.1% Tween 20 containing 5% non-fat milk) for 1 h, and then incubated with monoclonal mouse anti-human Cyclin B1 antibody (Santa Cruz Biotechnology, CA, USA, 1:200 dilution), polyclonal goat anti-human β-actin antibody (Santa Cruz Biotechnology, 1:2000 dilution) in blocking buffer overnight at 4°C. Then membranes were incubated with appropriate secondary antibody for 1 h at room temperature. The bound antibody was detected using an enhanced chemilumiscent (ECL) detection reagent (Santa Cruz Biotechnology) and the bands were scanned by Quantity One software (Bio-Rad Laboratories, Hercules, CA, USA). Normalized densities were determined with ratio of density of cyclin B1 to that of β-actin.

### Statistical analysis

All data were presented as mean ± SD. Student's *t*-test or One-way analysis of variance (ANOVA) were used to compare means. A *P *value less than 0.05 were considered statistically significant.

## Results

### Expression of cyclin B1 in human endometrium

**Figure 1:**
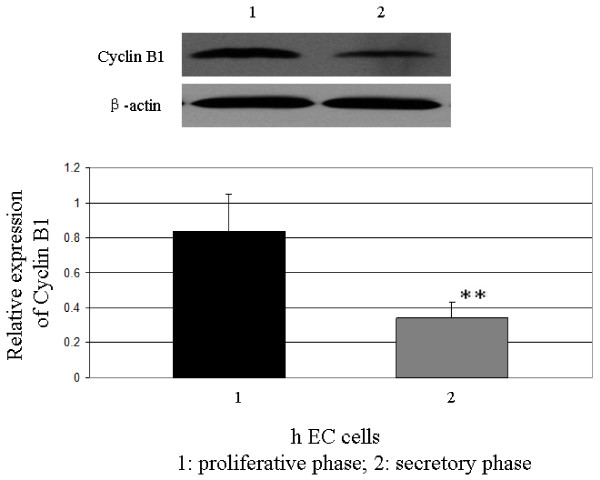
**Expression of cyclin B1 in human endometrium detected by Western blot**. The relative expression of cyclin B1 in human endometrium at the secretory phase is significantly lower than the proliferative phase (**P < 0.01).

12 pairs of human endometrium at proliferative phase and middle secretory phase were subjected to Western blot analysis. The result showed that the relative expression of cyclin B1 in human endometrium at the secretory phase is significantly lower than the proliferative phase (P < 0.01) (Figure 1).

### Progesterone inhibits growth of hECs

**Figure 2:**
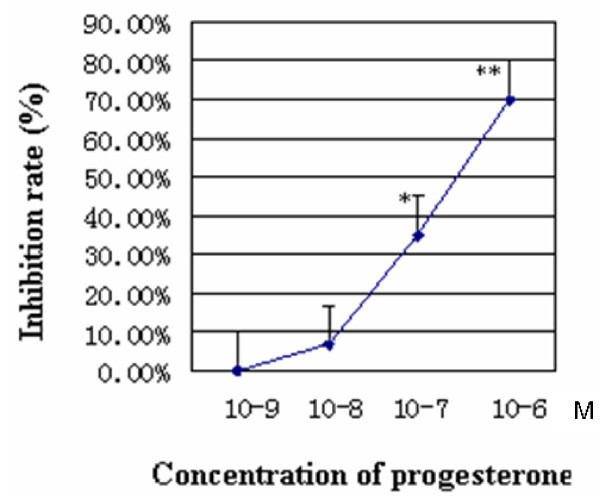
**Inhibition of cell growth on hECs by progesterone**. Human endometrial cells were treated with progesterone at concentrations of 1 × 10^-9^, 1 × 10^-8^, 1 × 10^-7 ^or 1 × 10^-6 ^M and cell growth was evaluated with MTT. Progesterone inhibited hECs growth in a dose-dependent manner. (*P < 0.05, **P < 0.01, compared with the control cells).

We examined the effect of progesterone on cell proliferation of primary hECs. Progesterone inhibited hECs growth in a dose-dependent manner. Progesterone did not inhibit hECs growth at the concentrations of 1 × 10^-9 ^M and 1 × 10^-8 ^M but significantly inhibited cell growth at 1 × 10^-7 ^M and 1 × 10^-6 ^M with inhibitory rates of 35.0% and 70.0% respectively (P < 0.05 and P < 0.01 respectively compared with control cells) (Figure 2).

### Progesterone and/or Alp decreases the expression of cyclin B1

**Figure 3:**
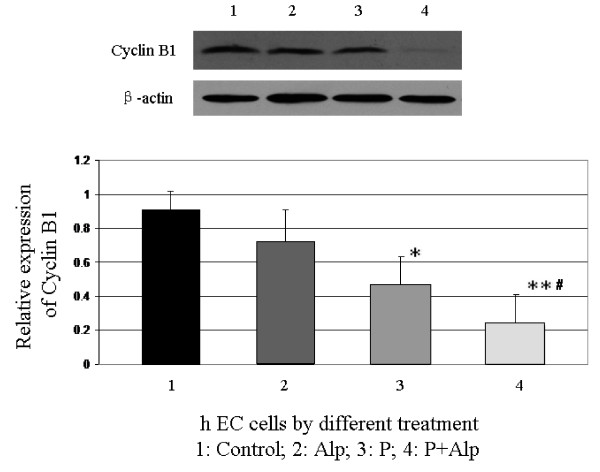
**Decreased expression of Cyclin B1 induced by progesterone and/or Alp**. Human endometrial cells were treated with progesterone and/or Alp. The expression of cyclin B1 was significantly decreased by the treatment of progesterone or progesterone and Alp, but not Alp alone (*P < 0.05, **P < 0.01, compared with the control cells). The levels of cyclin B1 was significantly decreased in cells treated with progesterone and Alp compared with those treated with progesterone or Alp alone (#P < 0.01).

The expression of cyclin B1 was significantly decreased by the treatment of progesterone at a concentration of 1 × 10^-7 ^M compared with control cells (P < 0.05), but not by the treatment of Alp alone at a concentration of 5 mM (P > 0.05). The levels of cyclin B1 was significantly decreased in cells treated with progesterone and Alp compared with those treated with progesterone or Alp alone or control cells (P < 0.01 for all) (Figure 3).

### Progesterone or plus Alp mediate cell cycle arrest at G2/M stage

**Figure 4:**
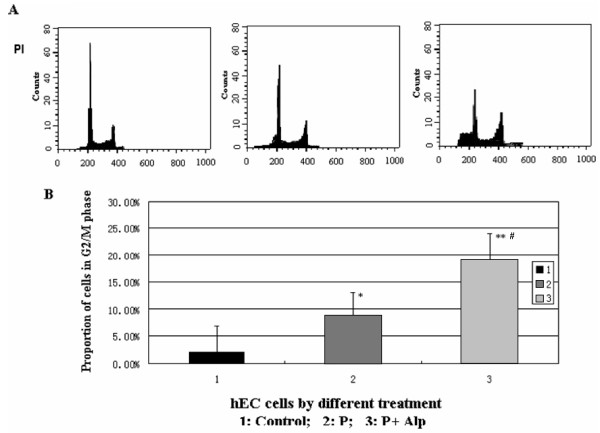
**Arrest of cell cycle in G2/M phase induced by progesterone and Alp**. Human endometrial cells were treated with progesterone alone or in combination with Alp. Cell cycles were analyzed by flow cytometry. A: Cell cycle analyzed with propidium iodide (PI) staining followed by flow cytometry. B: Comparison of the proportions of cells in G2/M phase. The proportion of cells in G2/M phase was significantly increased after hECs were treated with progesterone or progesterone and Alp (*P < 0.05, **P < 0.01, compared with the control cells). There was also significant difference between treatment with progesterone alone and progesterone plus Alp (#P < 0.05).

The proportion of cells in G2/M phase was significantly increased after hECs were treated with progesterone (P < 0.05), or progesterone and Alp (P < 0.01). 8.1% of cells were arrested in G2/M phase after the treatment of progesterone and 19.0% after the treatment of progesterone and Alp. There was also significant difference between treatment with progesterone alone and progesterone plus Alp (P < 0.05) (Figure 4).

### Progesterone or plus Alp induce apoptosis of hEC

**Figure 5:**
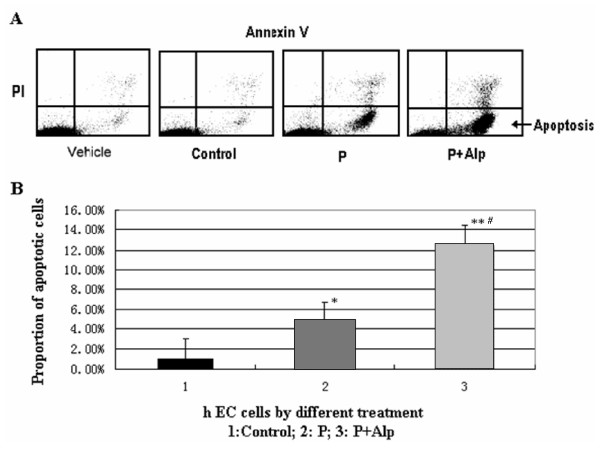
**Apoptosis of hECs induced by progesterone and Alp**. Human endometrial cells were treated with progesterone alone or in combination with Alp. Apoptosis was indexed by the detection of Annexin V. A: Apoptotic cells as labeled with Annexin V and followed by flow cytometry. B: Comparison of the proportions of apoptotic cells. The amount of apoptotic cells was significantly increased with the treatment of progesterone alone or progesterone plus ALP (*P < 0.05, **P < 0.01, compared with the control cells). There was also significant difference between treatment with progesterone alone and progesterone plus Alp (#P < 0.01).

The treatment with progesterone alone or progesterone plus ALP significantly increased the amount of apoptotic cells (P < 0.05 and P < 0.01 respectively compared with the control cells). The proportion of apoptotic cell was 4.7% after progesterone treatment and 12.5% after treatment of progesterone and Alp. There was also significant difference between treatment with progesterone alone and progesterone plus Alp (P < 0.01) (Figure 5).

## Discussion

In the present study, we demonstrated that the expression level of cyclin B1 in secretory endometria was significantly lower than in proliferative endometria. Progesterone inhibited the growth of hECs in a concentration-dependent manner. The treatment with progesterone significantly decreased the expression of cyclin B1, increased the proportions of cell in G2/M phase, and apoptotic cells. In addition, the presence of Alp enhanced the effects of progesterone on cyclin B1 down-regulation, G2/M cell cycle arrest and induction of apoptosis. Our results indicated that cyclinB1 played an important role in the endometrial cell cycle progression regulated by progesterone.

Cyclin B1, the master regulator in cell proliferation, plays an essential role in G2/M transition of mitosis in cell proliferation[19,20]. They are functionally involved in the rhythmic proliferation and differentiation of human endometrium and their actions are related to the levels of sex steroids in endometrium. Our experimental data confirmed the result of previous study that CyclinB1/Cdc2 was expressed in proliferating cells of the normal endometrium, and the expression of these molecules were suppressed in the secretory phase[17]. As the menstrual cycle is controlled by the sex steroids, mainly estrogen and progesterone, we speculated that the down-regulated expression of cyclin B1 in secretory endometrium is induced, at least partly, by the ascendant secretion of progesterone.

In vitro primary hECs culture confirmed the anti-proliferative effect of progesterone in endometrial cell. Our data showed that low dose of progesterone had no evident effect on the proliferation of hECs, but high dose of physiological level (10^-7 ^M) [21] and an even higher level (10^-6 ^M) could significantly inhibit the hECs proliferation. It is consistent with clinical application of large dose progesterone in treatment for endometrial proliferative disorders. As abundant expression of Cyclin B1 and alteration of the G2 pathway was reported in abnormal proliferative endometrial tissues, such as endometrial hyperplasia, endometrial adenocarcinoma and endometriosis[16,18,19,22-24] the down-regulation of cyclin B1 detected under the treatment of progesterone could well explain the underlying mechanism of the inhibitory effect of progesterone on endometrial cell growth.

A few previous studies reported that the growth inhibitory effect of progesterone in endometrial cell was induced by the enhancement of cell cycle arrest at the G1/S phase[25,26]. Recently, microarray analysis of progesterone effects on endometrial cell done by Paulssen et al[27] indicated the significantly down-regulation of cyclin B1. In these studies, either mice endometrial epithelial cell or human endometrial epithelial cancer cell line, Ishikawa cells, were used as cell model. In the current study, we used human primary endometrial cell for the treatment of progesterone, it was more close to the human physiology. Flow cytometry results showed that progesterone acted on cell cycle progression by regulating G2/M transition in hECs with the down-regulation of cyclin B1. We supposed that some of the different findings were due to the different cell models and progesterone types and doses used in the separate experiments. Results from primary human endometrial cell culture with physiological high level progesterone treatment in our experiment provided new evidence of the underlying mechanism.

It is considered that endometrium apoptosis was regulated by hormonal changes[28]. In the present study, apoptosis was observed in cultured hECs and the number of apoptotic cells was increased by the stimulation of progesterone, indicating high level progesterone induces apoptosis of endometrial cells. As G2/M arrest and apoptosis are common phenomena after genetic damage of the G2 pathway[23], we speculated that the induction of apoptosis in hECs by progesterone was also conducted by down-regulation of cyclin B1. This might be one of the mechanisms of anti-proliferation and endometrial atrophy induction in women who receives large dose progesterone treatment for endometrial proliferative disorders.

The activity of cyclinB1 could be inactivated by its inhibitors, resulting in cell cycle arrest. CyclinB1/cdc2 inhibitors, such as Camptothecin and Paclitaxel, have been used for the treatment of malignancies and have been demonstrated to be effective in limiting tumor cell growth by down-regulation of cyclinB1/cdc2[29-32]. Alsterpaullone (Alp) is a specific inhibitor of cyclin B1/cdc2, which inactivates cyclin B1/cdc2 complex and results in the arrest of cell growth by competitively inhibiting ATP to combine with the catalytic domain of cdc2[29,33]. Our results showed that the presence of Alp significantly enhanced the effects of progesterone on cyclin B1 down-regulation, G2/M cell cycle arrest and induction of apoptosis. It further confirmed the pivotal role of cyclin B1 in the progesterone active pathway in hECs. Previous studies showed that although Alp alone induced cell arrest at G2/M phase, the proportion was only 2-fold higher than control[29]. In the present study, the proportion of the G2/M cell cycle arrest induced by progesterone plus Alp was around 9-fold higher than control and 2.5-fold higher than progesterone only. The proportion of endometrial cell apoptosis induced by progesterone plus Alp was 12-fold higher than control and 3-fold higher than progesterone only. Our results suggest that Alp could enhance the inhibitory effect of progesterone on endometrial cell growth and apoptosis. Although further study is needed to clarify the mechanisms involved in these effects by Alp, these findings implicated that use of progesterone together with ingredient of cell cycle inhibitors might improve the therapeutic effect of hyperplasia, adenocarcinoma of endometrium and endometriosis. It will be interesting to validate this conjecture in the further research.

## Conclusion

In conclusion, progesterone may inhibit cell proliferation, mediate G2/M cell cycle arrest and induce apoptosis in hECs via down-regulating Cyclin B1. The presence of Alp enhanced the effects of progesterone on cyclin B1 down-regulation, G2/M cell cycle arrest and induction of apoptosis. Our findings suggest that cyclin B1 is a critical factor in proliferation and differentiation of hECs. Progesterone derivatives in combination with ingredient of CyclinB1 inhibitors may be a promising way for the treatment of endometrial proliferative diseases. Future investigations targeting the progesterone pathway on aberrant endometrial cell may be fruitful for developing a novel proposal of progesterone treatment.

## Competing interests

The authors declare that they have no competing interests.

## Authors' contributions

LT and YZ participated together with JZS and HFH in the design of the study. LT, YZ, HP and QL carried out the experiments. Data analysis was performed by XMZ and LT. The manuscript was written by LT and YZ. MYD, PCKL, JZS and HFH critically read the manuscript. All authors read and approved the final manuscript.
